# Massive Pancreatic Pleural Effusion Caused by Intrathoracic Perforation of a Pancreatic Pseudocyst Successfully Managed with Endoscopic Drainage Followed by Thoracoscopic Decortication: A Case Report

**DOI:** 10.70352/scrj.cr.26-0079

**Published:** 2026-04-21

**Authors:** Makoto Hirai, Hiroki Ebana, Kohei Tagawa, Aki Kobayashi

**Affiliations:** 1 Department of Thoracic Surgery, Kasukabe Municipal Medical Center, Kasukabe, Saitama, Japan; 2 Department of Thoracic Surgery, Tokyo Metropolitan Bokutoh Hospital, Tokyo, Japan

**Keywords:** pancreatic pleural effusion, pancreatic pseudocyst, thoracoscopic surgery, chronic pancreatitis

## Abstract

**INTRODUCTION:**

Pancreatic pleural effusion is a rare condition caused by direct leakage of pancreatic fluid into the thoracic cavity, resulting in massive pleural effusion. We report a case of massive pleural effusion due to intrathoracic perforation of a pancreatic pseudocyst successfully treated by thoracoscopic surgery.

**CASE PRESENTATION:**

A 36-year-old man with a history of chronic alcoholic pancreatitis presented with progressive dyspnea and was transported to Bokutoh Hospital. Chest radiography revealed a massive left pleural effusion with mediastinal shift to the right. Emergency thoracic drainage was performed, resulting in symptomatic improvement. Biochemical analysis of the pleural fluid showed markedly elevated amylase levels. Endoscopic retrograde cholangiopancreatography demonstrated contrast leakage from a pancreatic pseudocyst into the left thoracic cavity, leading to a diagnosis of intrathoracic perforation of a pancreatic pseudocyst. Endoscopic nasopancreatic drainage was performed, and subsequent imaging confirmed closure of the fistula. However, residual pleural effusion and impaired lung expansion persisted. Thoracoscopic decortication and drainage were therefore performed on day 17 of hospitalization. Intraoperatively, a large amount of black pleural fluid and thickened inflammatory pleura resembling empyema were observed. The postoperative course was uneventful, and the patient was discharged home on POD 37 without recurrence.

**CONCLUSIONS:**

When pleural effusion is observed in patients with chronic pancreatitis, pancreatic pleural effusion should be considered. Thoracoscopic surgery may be an effective treatment option, particularly in cases with persistent pleural inflammation after successful endoscopic management.

## Abbreviations

ENPD
: endoscopic naso-pancreatic drainage
ERCP
: endoscopic retrograde cholangiopancreatography

## INTRODUCTION

Intrathoracic perforation of a pancreatic pseudocyst is a rare complication of chronic pancreatitis and may lead to massive pleural effusion, known as pancreatic pleural effusion. This condition results from direct leakage of pancreatic juice into the thoracic cavity and often presents with respiratory symptoms rather than abdominal complaints.

Although initial management generally consists of conservative and endoscopic treatment aimed at closing the pancreaticopleural fistula, persistent pleural inflammation may necessitate surgical intervention. Thoracoscopic surgery has been reported to be a useful and minimally invasive option in selected cases.

Herein, we report a case of massive pancreatic pleural effusion caused by intrathoracic perforation of a pancreatic pseudocyst, successfully treated with thoracoscopic surgery following endoscopic pancreatic duct drainage.

## CASE PRESENTATION

A 36-year-old man with a history of chronic alcoholic pancreatitis and depression was transported to the emergency department of Bokuto Hospital because of progressive dyspnea. He had experienced shortness of breath for approximately 1 month, which gradually worsened and eventually limited his ability to ambulate.

On admission, his height was 170 cm and body weight was 49 kg. Vital signs were as follows: body temperature 36.0°C, blood pressure 125/98 mmHg, heart rate 120 beats/min, respiratory rate 28 breaths/min, and oxygen saturation 89% while receiving 10 L/min of oxygen via a reservoir mask.

**Fig. 1:**
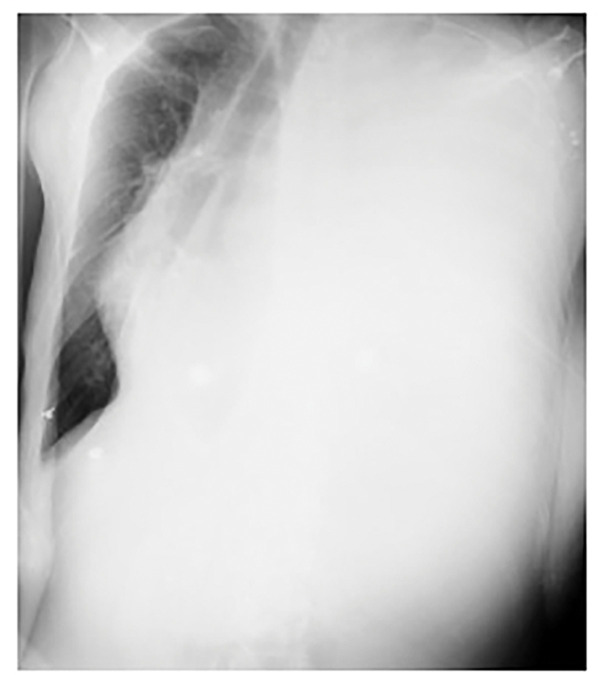
Chest radiograph at hospital admission. A plain chest radiograph at hospital admission showing massive left pleural effusion with mediastinal shift to the right.

A chest radiograph demonstrated massive left-sided pleural effusion with marked mediastinal shift to the right (**Fig. 1**). Emergency left thoracic drainage was immediately performed, resulting in rapid improvement of dyspnea. The patient had been followed by the gastroenterology department for chronic pancreatitis with a pancreatic pseudocyst. Given this history and the sudden onset of massive left pleural effusion, pancreatic pleural effusion was suspected, and pleural fluid amylase was measured. Biochemical analysis of the pleural fluid revealed a markedly elevated amylase level of 17903 IU/L.

**Fig. 2:**
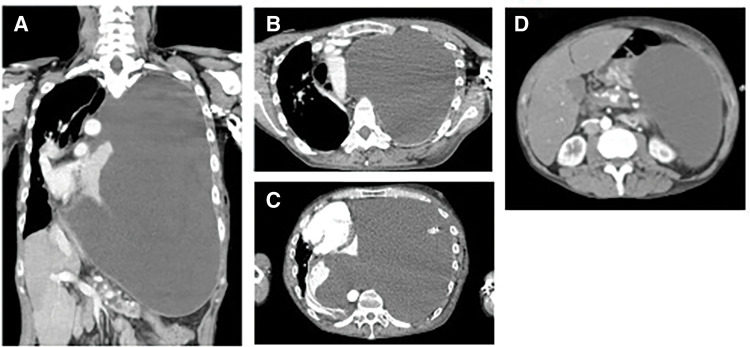
Contrast-enhanced CT scans at hospital admission. (**A**–**C**) CT images showing massive left pleural effusion and mediastinal shift to the right. No apparent communication between the pancreatic pseudocyst and the thoracic cavity is detected on CT. (**D**) Abdominal CT showing pancreatic calcifications consistent with chronic pancreatitis and a suspected pancreatic pseudocyst.

**Fig. 3:**
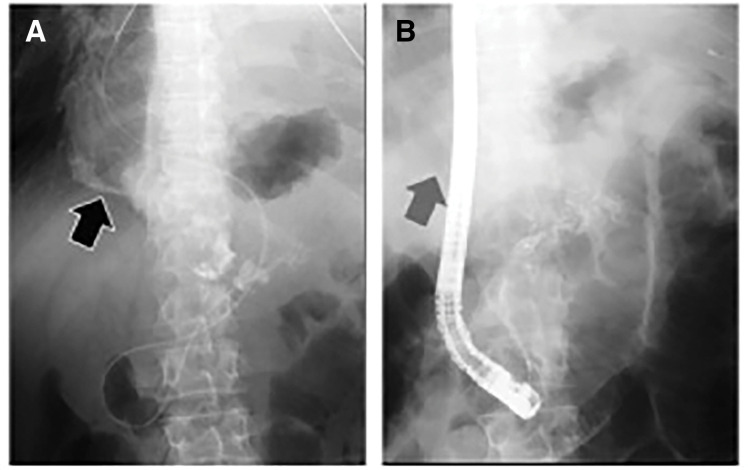
ERCP findings on hospital day 3 (**A**) and day 8 (**B**). (**A**) Contrast leakage from the pancreatic pseudocyst into the left thoracic cavity (black arrow). (**B**) Disappearance of the contrast leakage after ENPD (gray arrow indicates the previous leakage site). ENPD, endoscopic naso-pancreatic drainage; ERCP, endoscopic retrograde cholangiopancreatography

CT of the chest and abdomen showed massive left pleural effusion and mediastinal deviation. Abdominal CT also demonstrated a pancreatic pseudocyst associated with chronic pancreatitis; however, no obvious communication between the pseudocyst and the thoracic cavity was identified on CT (**Fig. 2**). On hospital day 3, ERCP demonstrated contrast leakage from a pancreatic pseudocyst into the left thoracic cavity (**Fig. 3A**). Other potential causes of amylase-rich pleural effusion, such as esophageal rupture or malignancy, were considered unlikely based on the clinical course and imaging findings. Based on these findings, intrathoracic perforation of a pancreatic pseudocyst was diagnosed.

**Fig. 4:**
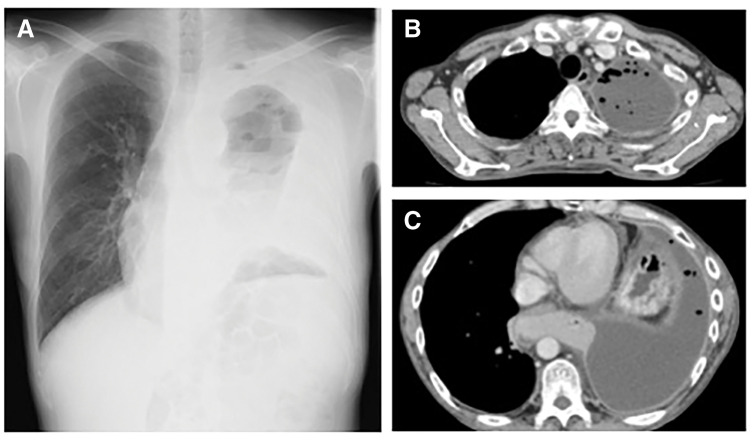
Imaging findings after naso-pancreatic drainage. (**A**) Chest radiograph showing persistent left pleural effusion with incomplete re-expansion of the left lung. (**B**, **C**) Follow-up contrast-enhanced chest CT demonstrating a loculated left pleural effusion with marked pleural thickening and incomplete re-expansion of the left lung, suggesting organized pleural inflammation and lung entrapment.

ENPD was performed to decompress the pancreatic duct. Follow-up ERCP on hospital day 8 confirmed disappearance of contrast leakage into the thoracic cavity (**Fig. 3B**). Despite successful closure of the pancreaticopleural fistula, residual pleural effusion, impaired lung expansion, and persistent oxygen requirement continued. Follow-up contrast-enhanced CT performed after confirmation of fistula closure revealed a loculated left pleural effusion with marked pleural thickening and incomplete re-expansion of the left lung (**Fig. 4**), suggesting organized pleural inflammation and lung entrapment.

**Fig. 5:**
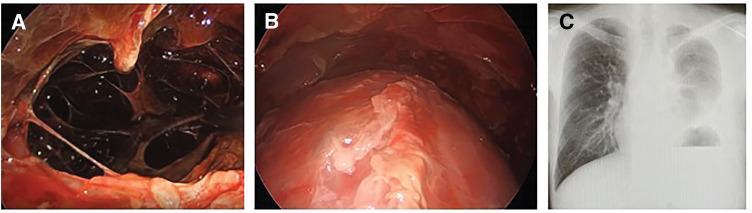
Intraoperative and postoperative findings. (**A**) Thoracoscopic findings showing a large amount of black pleural fluid and markedly thickened inflammatory pleura, consistent with empyema. (**B**) Thoracoscopic view after decortication showing satisfactory lung re-expansion before chest closure. (**C**) Chest radiograph at discharge showing good re-expansion of the left lung.

Because prolonged pleural inflammation due to pancreatic juice leakage was suspected, thoracoscopic surgery was indicated. On hospital day 17, thoracoscopic decortication and pleural lavage were performed under general anesthesia with single-lung ventilation, with the patient in the right lateral decubitus position. A 7-mm port was placed in the fifth intercostal space along the posterior axillary line, and a 2-cm utility incision was made in the fourth intercostal space along the mid-axillary line. Intraoperatively, a large amount of black pleural fluid with multiple septations and markedly thickened inflammatory pleura was observed, resembling empyema (**Fig. 5A**). The inflammatory pleura was removed as completely as possible, and the thoracic cavity was thoroughly irrigated with a large volume of sterile distilled water. After removal of the thickened pleura, satisfactory lung re-expansion was confirmed thoracoscopically (**Fig. 5B**). Two chest drains were then placed. The operative time was 1 h 53 min, and blood loss was 153 mL, excluding pleural fluid.

The postoperative course was uneventful. Oxygen supplementation was gradually tapered and discontinued. Pleural fluid amylase measured on POD 7 was 80 U/L. After the disappearance of air leakage and reduction of drainage volume to approximately 100 mL/day with a low pleural amylase levels, all chest drains were removed on POD 7 following confirmation of adequate lung re-expansion on chest radiography. The patient was discharged home ambulatory on POD 20. Postoperative chest radiography at discharge confirmed good re-expansion of the left lung (**Fig. 5C**). The patient has been followed in the gastroenterology outpatient clinic at Bokuto Hospital for approximately 4 years, with no recurrence of pleural effusion.

## DISCUSSION

Pancreatic pleural effusion is defined as a condition in which pancreatic juice directly leaks into the thoracic cavity, resulting in massive pleural effusion. It is thought to occur when increased pressure within the pancreatic duct leads to disruption of a pancreatic pseudocyst or the pancreatic duct itself, allowing pancreatic juice to pass through the retroperitoneal space and enter the thoracic cavity via the esophageal or aortic hiatus. This condition predominantly affects men in their 30s to 60s and occurs more frequently on the left side (57%), followed by the right side (29%) and bilaterally (14%).^1)^ Approximately 99% of cases of pancreatic pleural effusion develop in patients with alcoholic chronic pancreatitis.^2)^ The most common symptom is dyspnea, which is observed in approximately half of the patients.^3)^ Other reported manifestations include hemoptysis caused by bronchopleural fistula formation^4)^ and pericardial effusion resulting from fistulization to the pericardium,^5)^ indicating that pancreatic pleural effusion can present with a wide variety of clinical features.

For the treatment of pancreaticopleural fistula associated with pancreatic pleural effusion, conservative management aimed at fistula closure and suppression of pancreatic secretion is generally recommended as the initial approach. This includes fasting, nasogastric tube decompression, total parenteral nutrition, drainage using pancreatic duct stenting,^2)^ and administration of somatostatin analogs.^6)^ If conservative treatment fails after more than 3 weeks, surgical intervention should be considered, and pancreatic resection has been reported in approximately 10%–30% of cases.^7)^ In particular, pancreatic pseudocysts complicated by infection, obstruction, rupture, or hemorrhage require aggressive surgical management.^8)^

Pancreatic pleural effusion is a rare condition. In our search of the Japan Medical Abstracts Society database using the keyword “pancreatic pleural effusion” for the period from 2005 to 2024, a total of 40 cases reported in Japan were identified after excluding conference abstracts.

The median age of the patients was 54 years (range, 35–81 years), and 36 patients were male. The underlying disease was alcoholic chronic pancreatitis in the majority of cases (n = 34), followed by pancreatic tumors in 3 cases, postoperative abdominal conditions in 2 cases, and 1 case occurring after CT-guided biopsy of a pancreatic tumor. Regarding treatment, thoracic drainage was performed in 36 cases, pancreatic duct drainage in 30 cases, thoracic surgery in 10 cases, and abdominal surgery in 15 cases.

Similar to the present case, only 4 cases were treated with thoracic surgery after thoracic drainage and pancreatic duct drainage, and all of these cases were reported after 2013.^9–12)^ Furthermore, since 2013, only 2 cases of pancreatic pleural effusion caused by alcoholic chronic pancreatitis have been treated with abdominal surgery: 1 case complicated by infected pancreatic pseudocyst^13)^ and 1 case associated with severe biliary stricture.^7)^ This trend is considered to reflect improvements in endoscopic treatment techniques and increased accuracy of endoscopic pancreatic juice drainage procedures.^11)^

In the present case, the pancreaticopleural fistula resolved with fasting and decompression using an ENPD tube alone; therefore, somatostatin analogs were not administered. As there were no findings suggesting the need for abdominal surgical intervention as described above, abdominal surgery was deemed unnecessary. Consequently, surgical treatment was limited to thoracoscopic surgery to address inadequate pleural drainage and persistent oxygen requirement. Postoperatively, the oxygen requirement resolved, and the patient was discharged home ambulatory. No recurrence has been observed to date. In this clinical context, the chosen treatment strategy and timing of surgical intervention were considered reasonable.

Pancreatic resection is more invasive than thoracoscopic lavage and drainage. In addition, from the standpoint of preserving pancreatic function in patients with chronic pancreatitis, pancreatic resection should be avoided whenever possible. When closure of the pancreaticopleural fistula has been confirmed, surgical management limited to minimally invasive thoracoscopic surgery alone may be an effective treatment option.

## CONCLUSIONS

We reported a rare case of massive pancreatic pleural effusion caused by intrathoracic perforation of a pancreatic pseudocyst, successfully treated with thoracoscopic surgery following endoscopic pancreatic duct drainage. When pleural effusion is observed in patients with chronic pancreatitis, pancreatic pleural effusion should be considered, and thoracoscopic surgery may be a valuable treatment option.

## DECLARATIONS

### Availability of data and materials

All data generated or analyzed during this study are included in this published article.
